# 1346. Genetic Variation at the Interferon λ Locus and Association with Clinical Outcome and Severity of COVID-19

**DOI:** 10.1093/ofid/ofad500.1183

**Published:** 2023-11-27

**Authors:** Dana Alalwan, Riya Negi, Alejandro García-León, Gurvin Saini, Colette Marie Gaillard, Grace Kenny, Stefano Savinelli, Eoin R Feeney, Aoife Cotter, Michael Carr, Gabriel Gonzalez, Alan Landay, Eoghan de Barra, Obada Yousif, Mary Horgan, Patrick Mallon

**Affiliations:** Centre for Experimental Pathogen Host Research (CEPHR), University College Dublin, Belfield, Dublin, Ireland; 1Centre for Experimental Pathogen Host Research (CEPHR), University College Dublin, Belfield, Dublin 4, Ireland, Dublin, Dublin, Ireland; Centre for Experimental Pathogen Host Research (CEPHR), University College Dublin, Belfield, Dublin 4, Ireland, Dublin, Dublin, Ireland; 1Centre for Experimental Pathogen Host Research (CEPHR), University College Dublin, Belfield, Dublin 4, Ireland, Dublin, Dublin, Ireland; 1Centre for Experimental Pathogen Host Research (CEPHR), University College Dublin, Belfield, Dublin 4, Ireland, Dublin, Dublin, Ireland; 1Centre for Experimental Pathogen Host Research (CEPHR), University College Dublin, Belfield, Dublin 4, Ireland 2Department of Infectious Diseases, St Vincent’s University Hospital, Elm Park, Dublin 4, Ireland, Dublin, Dublin, Ireland; Centre for Experimental Pathogen Host Research (CEPHR), University College Dublin, Belfield, Dublin 4, Ireland, Department of Infectious Diseases, St Vincent’s University Hospital, Elm Park, Dublin 4, Ireland, Dublin, Dublin, Ireland; Centre for Experimental Pathogen Host Research (CEPHR), University College Dublin, Belfield, Dublin 4, Ireland, Department of Infectious Diseases, St Vincent’s University Hospital, Elm Park, Dublin 4, Ireland, Dublin, Dublin, Ireland; 1Centre for Experimental Pathogen Host Research (CEPHR), University College Dublin, Belfield, Dublin 4, Ireland 5Department of Infectious Diseases, Mater Misericordiae University Hospital, Eccles St, Dublin 7, Ireland, Dublin, Dublin, Ireland; National Virus Reference Laboratory, University College Dublin, Belfield, Dublin 4, Ireland, Dublin, Dublin, Ireland; National Virus Reference Laboratory, University College Dublin, Belfield, Dublin 4, Ireland, Dublin, Dublin, Ireland; 9Department of Internal Medicine, Rush University, Chicago, Il, USA, Chicago, Illinois; 6Department of Infectious Diseases, Beaumont Hospital, Beaumont, Dublin 9, Ireland 7Department of International Health and Tropical Medicine, Royal College of Surgeons in Ireland, Dublin, Ireland, Dublin, Dublin, Ireland; 4Endocrinology Department, Wexford General Hospital, Carricklawn, Wexford, Ireland, Wexford, Wexford, Ireland; 8Department of Infectious Diseases, Cork University Hospital, Wilton, Co Cork, Ireland, Cork, Cork, Ireland; University College Dublin, Dublin, Dublin, Ireland

## Abstract

**Background:**

People with obesity are at increased risk of severe COVID-19, with host inflammation a key contributor. Interferon (IFN) lambda 4 (IFNλ4) is a type III IFN expressed in individuals with the rs368234815-ΔG single nucleotide polymorphism (SNP) which is implicated in host immune responses to viral infections. We explored associations of this SNP to host inflammation, body mass index (BMI) and COVID-19 disease severity.

**Methods:**

Individuals with SARS-CoV-2, enrolled in the All-Ireland Infectious Diseases Cohort were genotyped for the rs368234815 SNP by allelic discrimination assay, and plasma circulating type I, II and III IFNs by immunoassay that were measured in a sub-cohort collected within ten days of symptom onset. We compared the prevalence of COVID-19 mild cases according to WHO criteria between IFNλ4 non-expressing (TT) and expressing (ΔG) genotypes using a Kruskal Wallis test. IFNλ4 polymorphisms affecting type I, II and III IFNs were assessed in the sub-cohort using a stepwise binary logistic regression adjusted for age, sex at birth, ethnicity, and comorbidities, including obesity (BMI ≥ 30 kg/m^2^).

**Results:**

853 participants were enrolled, of whom 471 (55%) harboured IFNλ4-TT/TT (Table 1). Expression of IFNλ4-ΔG was not significantly different between disease severity groups (P = 0.357). Within the early sampling sub-cohort (n = 321), we observed higher circulating IFNλ1 and IFNλ2 in those with more severe COVID-19 compared to mild disease (*P* < 0.01) (Fig 1). Only IFNλ2 remained significantly associated with mild COVID-19 in adjusted analyses (B COEFF 0.232 (0.067, 0.808), *P* = 0.021). We found no consistent associations between IFNλ4 genotypes and circulating interferons. Furthermore, we observed significantly higher IFNλ2 in people with obesity. However, the relationship between elevated plasma IFNλ2 and disease severity was only observed in people without obesity (*P* < 0.01) but not in those with obesity (Fig 2).

**Table 1**

**Figure:**
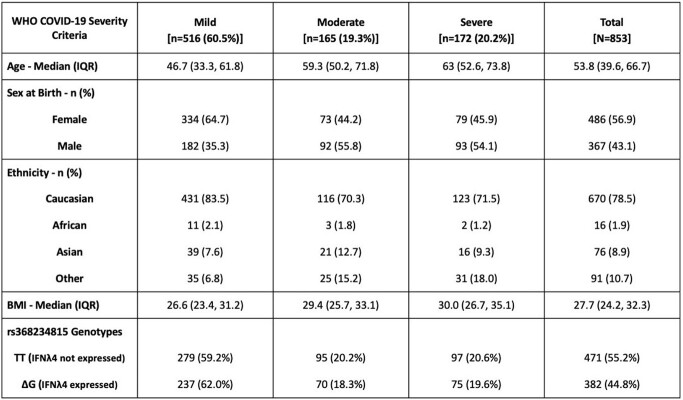

Clinicodemographic Details of Enrolled Patients in the AIID COVID-19 Cohort

**Figure 1**

**Figure:**
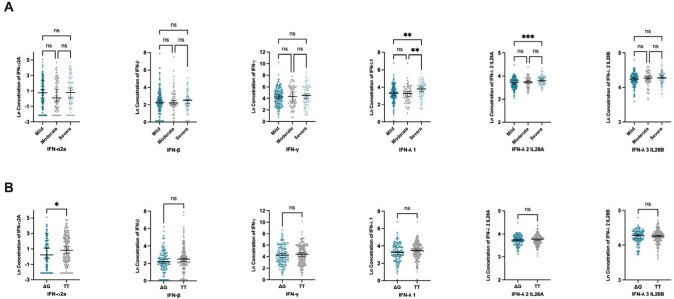

Plasma Circulating Levels (pg/mL) of Type I (IFN-α2a, and IFN-β), Type II (IFN-γ), and Type III (IFN-λ 1, IFN-λ 2 IL28A, IFN-λ 3 IL28B) Interferons. (A) World Health Organisation (WHO) COVID-19 disease severity criteria association with plasma circulating levels of type I, II, and III interferons. (B) Assessment of IFN-λ 4 expressing (ΔG) and non-expressing (TT) genotypes effects on circulating levels of type I, II, and III interferons. Ln - natural log; ns P > 0.05, * P ≤ 0.05, ** P ≤ 0.01, *** P ≤ 0.001.

**Figure 2**

**Figure:**
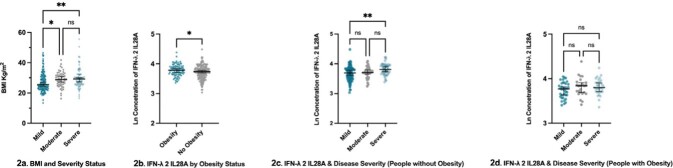

World Health Organisation (WHO) COVID-19 Disease Severity Association with Circulating Plasma Levels of Type III (IFN-λ 2 IL28A) Interferon and Obesity Status. Obesity defined as BMI ≥ 30 Kg/m2. Ln - natural log; ns P > 0.05, * P ≤ 0.05, ** P ≤ 0.01.

**Conclusion:**

IFNλ4 genotypes were not associated with COVID-19 disease severity or levels of circulating interferons. However, IFNλ2 was higher in individuals with obesity, though associations between IFNλ2 and disease severity was lost, suggesting that obesity may contribute to increased risk of severe COVID-19 through increased expression of IFNλ2.

**Disclosures:**

**All Authors**: No reported disclosures

